# Erectile Dysfunction in Chronic Kidney Disease and Hemodialysis Patients: A State-of-the-Art Review

**DOI:** 10.7759/cureus.79292

**Published:** 2025-02-19

**Authors:** Merkourios Kolvatzis, Apostolos Apostolidis, Fotios Dimitriadis, Konstantinos Hatzimouratidis, Kyriakos Moysidis

**Affiliations:** 1 2nd Department of Urology, Medical School, Aristotle University of Thessaloniki, Thessaloniki, GRC; 2 1st Department of Urology, Medical School, Aristotle University of Thessaloniki, Thessaloniki, GRC

**Keywords:** chronic kidney disease, erectile dysfunction, hemodialysis, renal transplantation, sexual dysfunction management

## Abstract

Erectile dysfunction (ED) is a prevalent condition that significantly impacts the quality of life and overall well-being of affected individuals. This state-of-the-art (SotA) review covers literature published between 2020 and 2024, focusing on adult patients with chronic kidney disease (CKD) at various stages, including those receiving hemodialysis and individuals who have undergone renal transplantation. The pathophysiology of ED in CKD patients is multifaceted, involving vascular, neurogenic, hormonal, and psychological components that are further exacerbated by the metabolic and hemodynamic alterations associated with kidney disease.

The review provides a comprehensive analysis of current diagnostic approaches, including clinical assessment tools, laboratory evaluations, and imaging modalities that aid in identifying the underlying causes of ED in CKD patients. Pharmacological treatments, particularly phosphodiesterase type 5 (PDE5) inhibitors, remain the cornerstone of therapy; however, their efficacy and safety in patients with renal impairment require careful consideration. Non-pharmacological interventions, such as lifestyle modifications, exercise regimens, and psychological support, are explored as integral components of a holistic management approach. Additionally, advanced therapeutic options, including intracavernosal injections, vacuum erection devices, penile prostheses, and emerging therapies such as low-intensity shockwave therapy, stem cell treatment, and gene therapy, are critically reviewed in the context of CKD.

This review also examines the bidirectional relationship between ED and comorbidities commonly associated with CKD, including cardiovascular disease, diabetes mellitus, and metabolic syndrome. Special emphasis is placed on the impact of dialysis modalities and renal transplantation on erectile function, highlighting the challenges in managing ED in these specific patient populations. Moreover, the psychological burden of ED, coupled with the stigma associated with CKD and sexual health, underscores the necessity of a multidisciplinary approach that integrates nephrologists, urologists, psychologists, and primary care providers.

Despite advancements in treatment modalities, challenges such as limited patient adherence, stigma, and disparities in healthcare access persist. Addressing these issues requires targeted educational initiatives and policy changes to enhance patient awareness and access to comprehensive care.

## Introduction and background

Introduction

Erectile dysfunction (ED) is the long-term inability to achieve or maintain an erection sufficient for satisfactory sexual performance and the grim reality faced by millions of men worldwide [1]. The prevalence of ED varies between 10% and 48% across different studies worldwide and is projected to affect a staggering more than 320 million people worldwide by 2025, a sharp increase from 152 million in 1995 [2]. It is strongly related to age and various comorbid conditions [3]. Its prevalence rises from 5% in men aged 20-39 to 14.8% in those 40-59 years old and reaches 70% in men over 70 [3] to nearly 80% at 80 years of age in men without comorbidities [4,5]. Among other aggravating vascular and neurogenic comorbid states that come with advanced age are hypertension, diabetes, atherosclerosis, hyperlipidemia, metabolic syndrome, and chronic kidney disease (CKD). The etiologies may include vascular and neurologic ED pathophysiologies, anatomical aberrations, hormonal abnormalities, side effects of medication, and psychological factors. This diminishes the quality of life of both the affected and their partner, and results in a tremendously burdensome cost to the world's economies. Several risk factors often occur together, making the diagnosis and therapy options more difficult [6-8].

CKD is also a major health issue worldwide, accompanied by significant morbidity and mortality due to its complications, ED being one of them [9]. CKD is defined as impaired kidney function, where the glomerular filtration rate (GFR) is below 60 ml/min/1.73 m², persisting for longer than three months [10]. Diabetes mellitus, hypertension, and numerous forms of glomerulonephritis have been identified as the principal causes of CKD [11]. While smoking plays a significant role in exacerbating CKD-related ED by contributing to vascular dysfunction and oxidative stress [12]. As CKD progresses to end-stage renal disease (ESRD), hemodialysis (HD), or peritoneal dialysis, renal replacement treatments, including kidney transplantation, may be needed [11]. ED is quite common among patients with CKD, especially among those who have been undergoing dialysis, with the rates in various studies ranging between 50% and 80% [12]. Understanding the multifactorial causes of ED in CKD is important in improving patients’ quality of life and building effective treatment strategies [11].

The pathophysiology of erectile dysfunction in CKD is relatively complex [12]. Testosterone levels decrease while there is an increase in plasma levels of luteinizing hormone (LH) and follicle-stimulating hormone (FSH) in patients with CKD [9]. However, ED may be the earliest clinical manifestation of diabetic autonomic neuropathy. Diabetes is actually the most common factor leading to end-stage CKD [13]. Peripheral neuropathies are common among CKD patients and affect both motor and sensory functions. Specifically, the affected patients suffer from numbness, burning pain, and diminished or absent tendon reflexes [14]. The autonomic nervous system may also be impaired, affecting blood pressure control and causing erectile dysfunction [15].

Abnormal carbohydrate, lipid, and protein metabolism is often recorded in CKD patients [11]. Further assisted by chronic inflammation accompanying CKD, this metabolic dysfunction places the uremic patient at particular risk of developing atherosclerosis [16]. This leads to ischemic coronary disease and, by extension, ischemia of penile arteries [17]. Diabetes and hypertension further "accelerate" atherosclerosis in dialysis patients, leading to the occurrence of vascular erectile dysfunction [18]. Most of them are also on medication for their cardiovascular comorbidities, mainly beta-blockers and diuretics, which often cause erection difficulties as a side effect [19,20]. However, replacing them with other medications does not really produce important improvements in erectile function [20].

The highly prevalent, reduced erythropoietin synthesis in patients with end-stage CKD promotes anemia, among multiple side effects, a reduction of oxygen flow to the corpora cavernosa. The end result is a reduction in nitric oxide synthesis and an increase in the production of contractile factors of endothelial origin, leading to increased smooth muscle tone and, thus potentially, injury to the erectile mechanism [21]. Finally, the psychosomatic component must not be ignored because anxiety, low self-esteem, and chronic fatigue are all features common to patients treated chronically with hemodialysis. These factors combine to result in reduced sexual interest [22].

The International Index of Erectile Function (IIEF) was developed alongside the clinical trials for sildenafil and has since become the standard tool for assessing treatment outcomes in ED clinical studies [23]. IIEF has two versions: the extended 15-item version (IIEF-15) [24] and the shortened 5-item version (IIEF-5) [25]. The questions in the IIEF-15 assess erection, orgasm, desire, satisfaction from sexual intercourse, and overall satisfaction. All questions concern the last four weeks [24].

Objective

This state-of-the-art (SotA) review aims to deliver the most updated insights into the latest advancements in the pathophysiology, prevalence, contributing factors, and management of ED in patients with CKD and those undergoing hemodialysis, by focusing on studies between 2020 and 2024. It also assesses existing management strategies, highlights promising new interventions, and identifies gaps where further research can improve clinical outcomes and overall patient well-being.

Scope

This SotA review covers literature published between 2020 and 2024, focusing on adult patients with CKD at various stages-including those receiving hemodialysis and individuals who have undergone renal transplantation. Topics of investigation range from hormonal, vascular, metabolic, and neurological mechanisms to evaluations of pharmacological and lifestyle-based treatments, surgical approaches, and novel therapies such as regenerative medicine and extracorporeal shockwave therapy. Attention is also given to psychosocial and cultural factors that influence ED, underscoring the importance of personalized care. Studies involving pediatric populations and non-English publications are excluded. Through this targeted scope, the review offers a focused perspective on current clinical practices while pointing to potential areas for future exploration.

Methodology

Inclusion and Exclusion Criteria

This SotA review examines recent research published since 2020, focusing on studies that utilized either the complete IIEF-15 or the short IIEF-5 versions to assess erectile dysfunction (ED). Relevant studies published in English were identified through comprehensive searches on PubMed and Google Scholar using keywords such as "erectile dysfunction," "chronic kidney disease," "hemodialysis," "prevalence," "pathophysiology," "management," and "International Index of Erectile Function (IIEF-15/IIEF-5)." Boolean operators (AND/OR) were applied to refine the search results.

Clinical studies that offered substantial insights into the pathophysiology, prevalence, or management of ED in the context of CKD or hemodialysis, ensuring that ED was clearly addressed as a primary or secondary outcome, were included.

Exclusion criteria encompassed investigations focusing on other populations, animal-model research, papers lacking original data (such as reviews or editorials), and any studies that were unrelated to CKD or did not employ validated ED assessment tools.

## Review

Overview of selected studies

This SotA review includes 17 clinical studies published between 2020 and 2024, encompassing 1,961 patients with CKD and those undergoing hemodialysis. The studies varied in design, including seven cross-sectional studies, three observational studies, three comparative observational studies, two case-control studies, one randomized controlled trial, and one retrospective study. Sample sizes ranged from 42 to 639 participants. Most of the studies utilized the IIEF-5 questionnaire to evaluate ED, though some employed the complete IIEF-15 and additional tools like the ADAM (Androgen Deficiency in the Aging Male) questionnaire and the Hospital Anxiety and Depression Scale (HADS).

Consistent findings across the studies indicated a high prevalence of ED in CKD and hemodialysis patients, with rates ranging between 47.6% and 70.8%. The severity of ED was frequently associated with the duration of CKD, the presence of comorbidities such as diabetes and hypertension, as well as psychosocial and hormonal factors. Notably, renal transplantation and peritoneal dialysis showed significant improvements in erectile function compared to hemodialysis. Pharmacological interventions, particularly with phosphodiesterase type 5 (PDE5) inhibitors like tadalafil and alprostadil, were shown to enhance erectile function scores effectively. Additionally, some studies proposed that ED could serve as an early clinical marker for CKD, underscoring its potential role in early detection and management strategies. The inclusion of these recent studies provides a comprehensive analysis of the prevalence, pathophysiology, and management approaches for ED in patients with CKD, offering valuable insights for both clinical application and future research directions.

Prevalence and impact of ED in CKD

Several recent studies have pointed to an increased prevalence of ED among CKD patients. Jarullah et al. (2020) conducted a cross-sectional study of male hemodialysis patients and found that 40/84 (47.6%) experienced ED [26]. In a recent single-center, non-interventional, cross-sectional observational study, Fu et al. (2024) examined a small sample of Chinese CKD patients and identified a 41/72 (56.9%) prevalence of ED [8]. In contrast, highlighting differences across nations, a Brazilian study reported a prevalence of 66.3% [27]. Barros et al. (2024), in a similarly sized quantitative cross-sectional study, associated ED prevalence with factors such as advanced age, low family income, diabetes, reduced mean corpuscular hemoglobin, elevated total calcium, and decreased albumin levels [27]. Interestingly, despite the findings from IIEF-5, 76% of respondents rated their sex lives as regular to excellent, emphasizing the need to consider cultural influences that might obscure the condition. To provide a global perspective, Pyrgidis et al. (2021) conducted a systematic review and meta-analysis, which found an overall pooled ED prevalence of 5149/7253 (71%) among patients with end-stage renal disease [28]. The prevalence was 5729/7253 (79%) in those undergoing hemodialysis and 5149/7253 (71%) in patients on peritoneal dialysis [28].

Updates on the pathophysiology of erectile dysfunction in CKD

Vascular Issues

Vascular issues are significant in CKD patients due to widespread endothelial dysfunction, which reduces nitric oxide availability and impairs vasodilation. Increased arterial stiffness and atherosclerosis further restrict penile blood flow. Bratsiakou et al. (2023) examined the link between ED and pulse wave velocity (PWV) in CKD patients in a preliminary study involving 69 patients across CKD stages I-V [29]. The study found that declining kidney function was associated with increased arterial stiffness and a higher prevalence of ED. Notably, PWV emerged as the sole predictor of ED, underscoring the critical role of vascular factors [29].

Hormonal Factors

Hormonal imbalances also play a major role in erectile dysfunction among CKD patients. Reduced testosterone levels, coupled with elevated LH and FSH, disrupt normal sexual function [9]. In CKD, hormonal imbalances disrupt the body’s natural mechanisms, leading to low testosterone levels while LH and FSH rise in a compensatory but ineffective response [9,30]. This imbalance, worsened by uremic toxins, chronic inflammation, and oxidative stress, not only affects overall well-being but also damages blood vessels and reduces nitric oxide availability, making it even harder to achieve or maintain an erection [16,31]. A case-control study by Ismail et al. (2021) assessed erectile function in CKD patients and found significantly lower free testosterone levels in CKD patients compared to healthy controls [30]. Furthermore, the severity of ED was notably higher in patients on hemodialysis than those receiving medical treatment alone, highlighting the hormonal influence on ED [30]. Another recent study by Maciel et al. (2023) focused on hypogonadism and ED in hemodialysis patients with CKD [31]. It showed that hypogonadism exacerbated sexual dysfunction, with these patients experiencing reduced libido and erectile difficulties earlier than those without hypogonadism [31].

Neurological Factors

Uremic toxins-induced neuropathy affects autonomic and peripheral nerves, impairing neural pathways related to erection. Duarsa et al. (2021) evaluated the degree of erectile dysfunction (ED) resolution in patients treated with regular hemodialysis and continuous ambulatory peritoneal dialysis (CAPD) [32]. The study found notable differences in ED improvement, with CAPD demonstrating better outcomes [32]. This suggests that the dialysis method may influence neurological factors that impact erectile function. While the CAPD group showed significant improvements in both ED degree and IIEF-5 scores, the HD group did not exhibit such changes. The improvement in ED degree and IIEF-5 score was significantly better in CAPD patients compared to those undergoing HD.

Metabolic Factors

Erectile dysfunction can also be negatively affected by anemia and oxidative stress, which are common in CKD. Reduced oxygen delivery to tissues due to anemia impairs nitric oxide synthesis. Hossam Arafat et al. (2023) investigated the relationship between hemodialysis and oxidative stress markers in patients with ESRD undergoing regular dialysis [33]. They observed a significant positive correlation between hemodialysis-related factors and oxidative stress markers. The authors hypothesized that increased oxidative stress may contribute to the development of ED in these patients [33].

Bi-directional Influence and Psychological Factors

The relationship between ED and CKD is multifaceted and potentially bi-directional. ED may increase the risk of CKD, as recent findings suggest that men with ED have a higher likelihood of developing CKD, even when other risk factors like hypertension or diabetes are accounted for [34]. This intriguing potential association warrants further research to uncover potential mechanisms. Conversely, CKD can worsen ED, especially when the disease persists over time. The longer CKD progresses, the more it disrupts vascular and hormonal systems critical for achieving and maintaining an erection, leading to a greater severity of ED, particularly in hemodialysis patients [35]. Supporting this, Cirakoglu et al. (2021) identified a higher prevalence of CKD in individuals with ED, as assessed using the IIEF, suggesting that ED might serve as an early clinical marker for CKD [36].

Psychological Factors

Psychological factors further complicate this interplay. ED in CKD patients can both arise from and exacerbate psychological challenges, creating a vicious cycle. On one hand, anxiety, depression, and low self-esteem, common among CKD patients, can significantly contribute to the onset and severity of ED. For instance, Sy et al. (2020) demonstrated that psychological distress, including anxiety 35/65 (53.8%) and fear of failure 18/65 (27.7%), was prevalent in hemodialysis patients with ED, underscoring the importance of incorporating psychological support into their treatment plans [37].

Conversely, the presence of ED can exacerbate psychological issues in CKD patients, negatively affecting their quality of life [38]. ED can strain relationships and elevate the risk of mental health disorders. Warli et al. (2023) found a significant correlation between the severity of ED and anxiety in patients undergoing CAPD and hemodialysis, highlighting the impact of ED on mental health [39]. This analytic observational study with a cross-sectional design published in 2023 illustrated a significant difference in IIEF-5 scores between patients undergoing HD and CAPD [39]. The authors also used HADS and found a strong correlation between anxiety disorders and ED, but not with depression in their sample. This reinforces the need for a holistic approach to treatment, addressing both the physical and psychological aspects of ED in CKD patients.

**Figure 1:**
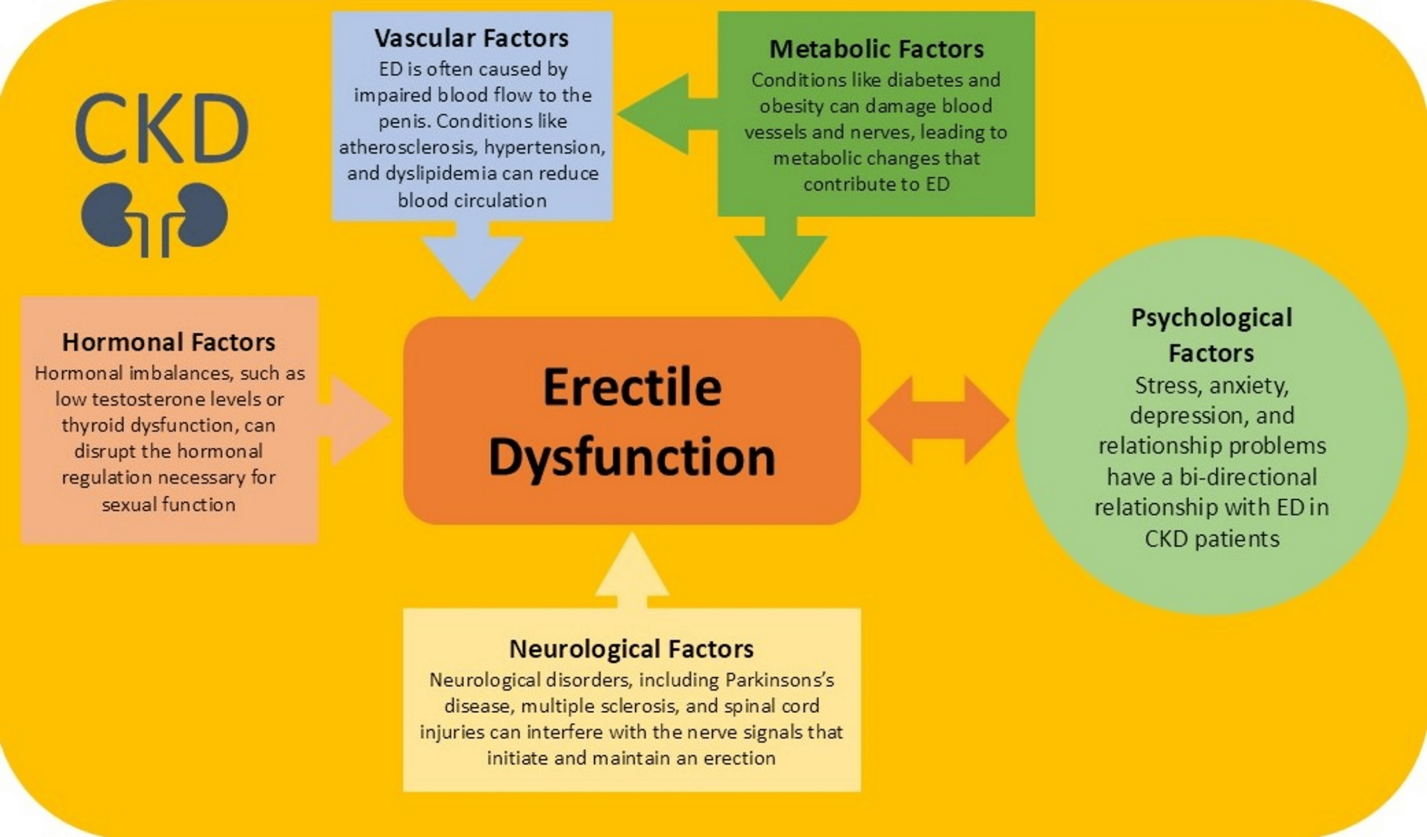
Factors affecting ED in CKD patients. ED, erectile dysfunction; CKD, chronic kidney disease. Image credit: Merkourios Kolvatzis.

Figure 1 showcases all the factors affecting ED in CKD patients.

Management and treatment options

Lifestyle Modifications and Psychological Support

Lifestyle interventions are instrumental in managing ED in CKD patients. Regular physical activity complementing a balanced diet, quitting smoking, and reduced or no alcohol can improve vascular health and erectile function [6]. Psychological support is equally important. Fryckstedt et al. (2023) used a modified IIEF questionnaire without scoring to assess ED and concluded that patients with CKD report deteriorating sexual function over time, regardless of treatment modality [40].

Pharmacological Treatments

PDE5 inhibitors, such as sildenafil and tadalafil, are commonly used to treat ED [6]. However, their effectiveness in CKD patients is variable. The efficacy of sildenafil in hemodialysis patients and post-renal transplant patients with persistent ED was very recently explored [41]. The study found that sildenafil was effective in 40.9% of HD patients and 76.9% of post-transplant patients [41]. The response was better in younger post-transplant patients with moderate to severe ED and shorter dialysis duration.

Testosterone replacement therapy may also be considered for patients with confirmed hypogonadism. Ismail et al. (2021) in their case-control study of 40 hemodialysis patients, 40 pre-dialysis patients, and 20 healthy controls, found that free testosterone levels were significantly reduced in CKD patients [30]. Also, they reported that the hemodialysis group had a statistically significant relationship between age and ED severity, thus they suggested that hormonal therapy could benefit those with low testosterone levels.

Non-pharmacological and Surgical Treatments

For patients unresponsive or not fit for pharmacological treatments, other options available include vacuum erection devices, intracavernosal injections, and penile implants [42]. Dell’Atti (2024) reviewed current treatment options for ED in kidney transplant recipients and found that extracorporeal shockwave therapy (ESWT) produced positive short-term effects on erectile function in patients who did not respond to first-line treatments [43]. Penile implants should be considered as a third-line option based on patient needs and clinical conditions [43]. 

Impact of Renal Transplantation

Renal transplantation can significantly improve ED in CKD patients by restoring normal kidney function and correcting hormonal and metabolic imbalances. Shoaab et al. (2020) conducted a comparative study between male renal transplant recipients and ESRD patients on hemodialysis [44]. They found that successful kidney transplantation significantly improved ED, especially in individuals with a shorter time on dialysis. Changes in sex hormone levels may contribute to this improvement. Similarly, Mondal et al. (2022) reported that sexual function in early middle-aged recipients improved in the majority of patients, after renal transplantation, with an overall significant improvement in all five domains of IIEF-15 [45].

By contrast, Spirito et al. (2020) found that kidney transplantation had a negative impact on sexual health, worsening both erectile and ejaculatory functions [46]. Factors such as smoking, age, diabetes, hypertension, pre-transplantation testosterone levels, and sexual function before transplant were significantly related to post-transplant sexual function.

Miron et al. (2023) assessed ED in renal transplant patients and found that the prevalence of sexual dysfunction increased with age, with only a small portion of their younger sample exhibiting normal sexual function [47]. They concluded that individualized assessment and management are crucial to addressing persistent ED in this population [47].

Efficacy of sildenafil and other agents in CKD and ESRD Patients

During the last few decades, sildenafil has been a cornerstone treatment for ED in patients with CKD and ESRD because of its favorable safety profile and effectiveness [48]. In the past five years, research has continued to validate its use among these patients, with studies consistently demonstrating its efficacy [49]. The major breakthrough in recent years lies in the growing evidence supporting its use even in dialysis patients, a group traditionally considered at high risk for cardiovascular events. Studies by Shoaab et al. (2020) and Jabali et al. (2020) have illustrated that even in complex cases, such as those involving renal transplantation or long-term hemodialysis, sildenafil remains effective and well-tolerated [44,50].

Other agents are also showing promise in the management of ED in CKD. Ozgur and Keseroglu utilized low-dose tadalafil and the IIEF questionnaire to assess changes in erectile function among hemodialysis patients, reporting significant improvements in the experimental group [51]. Similarly, Zakher et al. (2023) compared oral sildenafil, oral vardenafil, oral tadalafil, and intracavernosal injections of alprostadil among CKD patients [52]. They found that IIEF scores across all treatment groups were higher after therapy, with intracavernosal injections of alprostadil showing the highest mean scores among the modalities compared. However, both studies were relatively small, and a more rigorous approach in larger clinical settings is recommended. All identified and relevant clinical studies are included and summarized in Table 1.

**Table 1:** Studies from 2020 to 2024 on erectile dysfunction in patients with chronic kidney disease, including those on hemodialysis and post-renal transplant. IIEF, International Index of Erectile Function; ED, erectile dysfunction; CKD, chronic kidney disease; ADAM, Androgen Deficiency in the Aging Male; HADS, Hospital Anxiety and Depression Scale; CAPD, continuous ambulatory peritoneal dialysis; PWV, pulse wave velocity; PDE5, phosphodiesterase type 5.

Study	Study type	Sample size	Questionnaire type		Findings
Jarullah et al. (2020) [26]	Single-center cross-sectional	84	IIEF-5	47.6% of male hemodialysis patients had ED; no correlation with psychological factors.	
Ismy et al. (2020) [35]	Cross-sectional	60	IIEF-5	70% of CKD patients on hemodialysis had ED, severity worsened with CKD duration.	
Shoaab et al. (2020) [44]	Comparative observational	50	IIEF-5	Renal transplant patients showed significant improvement compared to those on hemodialysis.	
Jabali et al. (2020) [50]	Cross-sectional	59	IIEF-5	Most renal transplant patients improved erectile function post-transplant.	
Sy et al. (2020) [37]	Descriptive prospective	65	IIEF-5	70.8% of hemodialysis patients had ED, with notable psychosocial and hormonal impacts.	
Çırakoğlu et al. (2021) [36]	Retrospective	639	IIEF-5	ED may be an early clinical marker for CKD; severe ED correlated with early-stage CKD.	
Duarsa et al. (2021) [32]	Comparative observational	44	IIEF-5	CAPD patients showed better improvement in ED than hemodialysis patients.	
Ismail et al. (2021) [30]	Case-control	60	IIEF-5	High ED prevalence, especially severe in hemodialysis patients.	
Ozgur & Keseroglu (2022) [51]	Randomized controlled trial	58	IIEF-15	Tadalafil (5 mg/3 days) significantly improved all IIEF domains in hemodialysis patients.	
Mondal et al. (2022) [45]	Cross-sectional	135	IIEF-5	Significant improvement in sexual function post-renal transplant.	
Bratsiakou et al. (2023) [29]	Cross-sectional observational	69	IIEF-5	ED found in 52% of CKD patients; worsening kidney function linked to arterial stiffness (PWV).	
Maciel et al. (2023) [31]	Case-control	60	IIEF-5, ADAM	60% of hemodialysis patients had ED; hypogonadism also assessed.	
Warli et al. (2023) [39]	Cross-sectional analytical	42	IIEF-5, HADS	CAPD patients had higher IIEF-5 scores, indicating better erectile function than hemodialysis.	
Miron et al. (2023) [47]	Observational	170	IIEF-5	ED prevalence increased with age in kidney transplant patients; certain medications linked to ED.	
Fryckstedt et al. (2023) [40]	Observational	234	Custom IIEF-5 Questionnaire	CKD patients reported reduced sexual function, varying by treatment modality and CKD stage.	
Zakher et al. (2023) [52]	Comparative observational	60	IIEF-15	PDE5 inhibitors significantly improved erectile function scores among CKD patients. Intracavernosal injection of alprostadil provided the highest IIEF scores.	
Fu et al. (2024) [8]	Cross-sectional	72	IIEF-5	High prevalence of ED in CKD patients, with severity linked to diabetes and hypertension.	

Limitations

While considerable efforts have been made to understand ED in individuals with CKD and those undergoing hemodialysis, notable gaps are still evident. Many existing studies rely on cross-sectional methods, which complicates the ability to infer causality, and small sample sizes combined with diverse patient groups limit how these findings can be applied in the broad patient community [7,8]. Differences in age, various comorbid conditions, and differing treatment strategies further hinder the development of clear, standardized conclusions [28].

Additionally, while instruments like the IIEF-5 are commonly used, they are not always applied in a consistent manner, leading to discrepancies in data interpretation [23]. Some researchers have examined medications such as alpha-blockers and aspirin in relation to ED, but definitive evidence is lacking and underscores the need for prospective trials [19,20].

Moreover, psychosocial and cultural influences on ED remain underexplored, limiting opportunities to customize treatments to individual patient needs [38]. Emerging therapies like extracorporeal shockwave therapy and regenerative medicine have shown potential, yet they lack large-scale clinical trials to confirm their efficacy [42,43]. Research on long-term therapeutic outcomes is similarly sparse, as is understanding whether ED may serve as an early indicator of CKD [34].

To address these limitations will require multidisciplinary investigations to refine treatment approaches and optimize patient care. Such efforts are vital for improving the quality of life among those managing both CKD and ED.

Future directions

ED in CKD and hemodialysis patients is a complex condition requiring a multilevel therapeutic approach. Recent studies, from 2020 onwards, have advanced our understanding of the pathophysiological mechanisms and emphasized the need to address vascular, hormonal, neurological, metabolic, and psychological factors while highlighting the bidirectional cause-and-effect relationship between ED and CKD. Future research should focus on personalized treatment strategies, early detection of ED in CKD patients, and the development of therapies targeting oxidative stress and endothelial dysfunction. Additionally, investigating the impact of different dialysis modalities on ED and exploring new interventions like ESWT could further enhance patient outcomes. However, despite these advancements, the treatment landscape for ED in CKD and ESRD patients has not experienced a significant breakthrough beyond the established efficacy of PDE5 inhibitors like sildenafil. The ongoing challenge lies in effectively managing ED in these patients while simultaneously addressing underlying conditions that contribute to sexual dysfunction, such as cardiovascular disease, and diabetes, and the medications required to manage these comorbidities. Future therapies will likely need to focus on individualized treatment plans that integrate pharmacological, lifestyle, and psychological interventions to achieve optimal outcomes for patients.

## Conclusions

In conclusion, through comprehensive assessments and personalized treatment strategies, it is possible to improve erectile function and enhance the quality of life of these patients. Clinicians should adopt a holistic approach, incorporating lifestyle modifications, pharmacological treatments, and psychological support, as each element is equally essential in treating this condition.
